# Diabetes incidence and projections from prevalence surveys in Samoa over 1978–2013

**DOI:** 10.1007/s00038-017-0961-x

**Published:** 2017-03-09

**Authors:** Sophia Lin, Take Naseri, Christine Linhart, Stephen Morrell, Richard Taylor, Stephen T. Mcgarvey, Dianna J. Magliano, Paul Zimmet

**Affiliations:** 1 School of Public Health and Community Medicine, University of New South Wales, Sydney, Australia; 2 Ministry of Health, Apia, Samoa; 3 International Health Institute, Brown University, Providence, Rhode Island USA; 4 Baker IDI Heart and Diabetes Institute, Melbourne, Australia

**Keywords:** Developing Country, Incidence, Pacific Islands, Prevalence, Samoa, Type 2 diabetes

## Abstract

**Objectives:**

This study estimates type 2 diabetes (T2DM) incidence in Samoans aged 25–64 years from sequential, irregularly spaced, cross-sectional population prevalence surveys.

**Methods:**

T2DM prevalence from eight population surveys conducted over 1978–2013 (*n* = 12,516) was adjusted for census region, sex, and 5-year age group to the nearest previous census. Annual T2DM incidence was calculated from adjusted prevalences (by sex), using birth cohorts constructed from age-period matrices. Projections of T2DM incidence to 2020 were estimated, based on various scenarios of population weight change using Poisson regression.

**Results:**

Over 1978–2013, T2DM incidence was estimated to increase from 1.12 to 8.44 per 1000 person-years in men and from 2.55 to 8.04 per 1000 in women. Based on regression modeling, if mean population weight was stabilized from 2013, absolute incidence reductions of 0.9 per 1000 person-years (7% lower) are predicted in 2020, compared to the current period trend in weight gain.

**Conclusions:**

T2DM incidence can be calculated from irregularly conducted population risk factor surveys which may be useful in developing countries with limited resources.

**Electronic supplementary material:**

The online version of this article (doi:10.1007/s00038-017-0961-x) contains supplementary material, which is available to authorized users.

## Introduction

Incidence is the number of new cases of disease occurring in a population over a specified period. Estimation of incidence is preferable to prevalence, as prevalence does not distinguish between changes in the emergence of new cases contributing to the prevalent pool and changes in survival (prevalent cases remaining in the pool longer). An example from the Danish medication registry demonstrated that the increase in T2DM prevalence was due mainly to increased survival, not from increased incidence rates (Stovring et al. 2003). Effects of changes in population risk factors are detected earlier using incidence trends than prevalence changes, and incidence is not affected by changes in survival.

Incidence is usually measured using cohort studies, but these are subject to several problems. Cohort studies may: be arduous and expensive to perform, and can be affected by differential attrition bias; involve selection bias if generalisability is required and the sample is not representative; and involve Hawthorne effects in participants undergoing repeated re-interview leading to measurement bias (McCambridge et al. 2014). To estimate type 2 diabetes mellitus (T2DM) incidence in populations, other methods have also been employed, including: enumeration of cases through diabetes medication registries [such as in Australia (Australian Institute of Health and Welfare 2014)]; from repeat cross-sectional surveys of self-reported, newly diagnosed T2DM, such as in the United States (US) (Geiss et al. 2006); or from compartment modeling, such as the WHO DisMod program (Barendregt et al. 2003), or the statistical method proposed by Brinks and Landwehr (2015) based on prevalence, but also requiring accurate mortality data. However, these methods exclude those with undiagnosed T2DM (e.g., repeat cross-sectional surveys that rely on self-report only), exclude people diagnosed with T2DM who do not take medication (e.g., medication registries), while compartment models require cause-specific mortality data that often are not accurately recorded in many low resource countries. Novel methods of calculating T2DM incidence with wide applicability are thus needed.

Trends in the prevalence of T2DM have been calculated previously in Samoa (Lin et al. 2016a), but long-term trends in T2DM incidence are not known. From the single Samoan cohort study, of 29–60 year adults conducted between 1991 and 1995 (McGarvey 2001), 4-year incidence was estimated to be 1% (29–43 years) and 4% (44–60 years) in each sex, equivalent to an annualised incidence rate of 5.3 per 1000 person-years (29–60 years), after age adjustment to the 1991 population census (Samoa Department of Statistics 1991). Before and since this cohort study, several cross-sectional risk factor surveys have been conducted in Samoa to determine T2DM prevalence, but other incidence studies have not been undertaken.

In 1969, Stýblo et al. (1969) demonstrated that it is possible to use recurrent prevalence surveys to estimate (aggregate) annual incidence rates from birth cohorts derived from an age-period (Lexis) matrix of tuberculosis infection from recurrent population Mantoux surveys. Application of this method to calculation of T2DM incidence bypasses many of the weaknesses associated with current methods of calculating T2DM incidence, and has been previously demonstrated in Fijian adults (Morrell et al. 2016) where age-adjusted incidence was estimated to have increased from 2.6 to 5.0 per 1000 person-years over 1980–2011.

In the present study, eight risk factor prevalence surveys of T2DM conducted between 1978 and 2013 in Samoan adults aged 25–64 years were used to estimate incidence, with projections to 2020 under different population obesity scenarios using BMI as a predictor variable. T2DM incidences calculated from age-period matrices in the present study are externally validated by comparing to T2DM incidences calculated from a previously conducted empirical cohort study (McGarvey 2001).

## Methods

### Survey selection

Unit records from eight population NCD risk factor surveys were included in this analysis (*n* = 10,554): the Non-Communicable Disease Risk Factor (NCDRF) surveys of 1978 (*n* = 1079) (10) and 1991 (*n* = 1539) (Zimmet et al. 1981; Collins et al. 1994); the Samoan Adiposity and Cardiovascular Disease Risk Factor (SACRF) longitudinal study conducted in 1991 (*n* = 748) and 1995 (*n* = 719) (McGarvey 2001); the WHO Samoa STEPS surveys in 2002 (*n* = 2554) (WHO 2008) and 2013 (n = 1725) (WHO 2014); the Samoan Family Study of Overweight and Diabetes (SFSOD) of 2003 (*n* = 684) (DiBello et al. 2009); and the 2010 Genome-Wide Association Study (GWAS), *n* = 3468 (Hawley et al. 2014). Other risk factor surveys were not included in this analysis as they were conducted in a single village and could not be made nationally representative. For the present analysis, only non-pregnant adults aged 25–64 years who had a confirmed fasting status for plasma glucose (FPG) measurement were included.

### T2DM prevalence by 5-year age and period

T2DM has been defined as FPG ≥ 7.0 mmol/L and/or on medication for T2DM, or equivalent. A summary of the T2DM designation methods is available in the Online appendix; full methods of T2DM designation have previously been published (Lin et al. 2016a).

For each sex and 5-year age group, prevalences for each survey were calculated after being case weighted for census region, age group, and sex to the nearest previous census to minimise selection bias and improve the national representativeness at the time the survey was conducted. Case weights were calculated by dividing the proportion of the census sub-group population by the equivalent sub-group proportion in the risk factor survey, and adjusted prevalences from each survey were arranged into age-period matrices to calculate T2DM incidence.

### T2DM incidence

Stýblo et al. (1969) previously described a method for estimating annual incidence of tuberculosis infection from sequential prevalence surveys of Mantoux positivity. This birth cohort method was adapted to calculate annual T2DM incidence from sequential surveys of T2DM prevalence in Fiji, along with full details of the adapted method (Morrell et al. 2016). The method begins with prevalence estimates of the disease for a given age and period, and hence year of birth. The mean annual probability of *not* acquiring the disease, from year of birth to the given age for a given observation year, is then estimated from this prevalence. Then, for each given age, a straight line is fitted through a mathematically convenient transformation of this mean probability, as a function of the year of observation. The regression β-estimate for this trend is used in conjunction with the observed prevalence for the given year and age to estimate the cumulated probability of not having the condition by that age and year. One minus this quantity then becomes the estimate of incidence occurring for the given year and age.

Annual age-specific incidence estimates for each sex were directly age-standardized (Armitage et al. 2001) to the 25–64 year population in the 2011 Samoan census (Samoa Bureau of Statistics 2011), using 5-year age groups, separately by sex, and projected to 2020. Binomial methods were used to calculate 95% confidence intervals (95% CI) for age-specific and age-standardized incidences in each survey (Armitage et al. 2001).

For years between unevenly spaced surveys, interpolated denominator survey populations were calculated using adjacent surveys. Incident T2DM counts for each survey year were derived by multiplying calculated incidence rates by the survey population for each sub-group. Cumulative risk (%) across age groups for each survey was calculated as *[1 − exp(−x)] × 100*, where *x* is the cumulative incidence rate, derived from the sum over each age group *i* of the age-specific incidence rates *r*, times the number of ages in each age interval *n*: *Σ[r(i) × n(i)]* (Day 1992).

To assess the effect of period and birth cohort on T2DM incidence, Poisson regression models were used, with counts of incident T2DM as the outcome organised into 5-year age and period matrices and 5-year age and birth cohort matrices, for each sex, with the corresponding sample population for each stratum. Counts of T2DM incidence were modeled as a null fit, then with age alone, and further modeled with period or birth cohort to assess effect of period and birth cohort on T2DM incidence trends separately after adjusting for age. The difference in deviances between models was compared.

### Projections of T2DM incidence based on obesity scenarios

The previous projections of T2DM prevalence in Samoa have been based on period trends (Lin et al. 2016a), but no predictive projection modeling including BMI has been undertaken. Survey year (*n* = 7) and 5-year age group (*n* = 8) were arranged into 56 strata for each sex. Mean BMI, annual counts of incident T2DM, and populations were calculated for each stratum. Height and weight were measured in each included survey to enable calculation of BMI. Various Poisson models were developed for incidence modeling, and those with the lowest Akaike Information Criterion which produced a reasonable result were used for projection. For sex-specific T2DM incidence, annual incident counts were modeled using Conway–Maxwell Poisson regression with sex, age group, and mean BMI as explanatory variables. Survey year was not included in the regression models due to collinearity with mean BMI. Conway–Maxwell Poisson models were employed because of under-dispersion of regressor variables (Shmueli et al. 2005).

T2DM incidence estimates were then re-modeled and projected to 2020 according to several population weight change scenarios using BMI: (1) current mean population weight continues to increase at the current rate—to the previously predicted 2020 mean BMI (Lin et al. 2016a); (2) mean population weight is maintained at 2013 levels (i.e., no weight or BMI gain); and (3) mean population weight is reduced or increased by 1 kg steps to ±4 kg (with consequent changes in BMI). A ± 1–4kg weight change was selected as this represents a feasible percentage body weight change of ±1–5% both in men (mean weight in 2013 was 93.6 kg) and in women (mean weight in 2013 was 90.7 kg) (WHO 2014). 2013 was chosen as the baseline year as this is the most recent year for which nationally representative empirical population survey data are available.

Data were analysed using SAS 9.4 (SAS Institute Inc., Cary, NC, USA), SPSS 22 (IBM Corp., Armonk, NY, USA), and Microsoft Excel (Microsoft, Redmond, WA, USA).

## Results

**Fig. 1:**
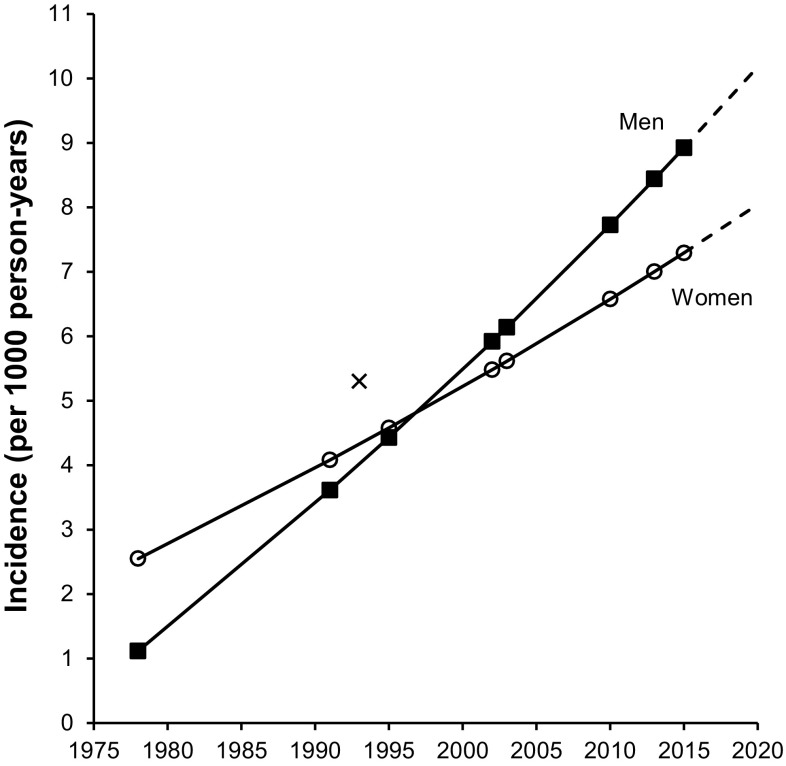
Estimated type 2 diabetes (T2DM) incidence (per 1000 person-years) in adults aged 25–64 years in Samoa for surveys conducted over 1978–2013, and projection to 2020. *Markers* indicate survey years. *Solid line* interpolated and modeled incidence using the Stýblo birth cohort method; *broken line* projected incidence. *X* indicates annual T2DM incidence for men and for women (5.3 per 1000 person-years) calculated over 1991–1995 from a cohort study by McGarvey (2001)

Over 1978–2013, annual national age-standardized T2DM incidence was estimated to increase from 1.74 to 7.25 per 1000 person-years (Table 1; Fig. 1). Based on the Samoan 25–64 year populations in the 1976 (42,639) and 2011 (71,968) censuses, this is equivalent to an increase from 75 new cases diagnosed in 1978 to 522 new cases diagnosed in 2013. Incidence was higher in women than in men, but the rate of increase has been faster in men, with incidence exceeding women from 1997.

**Table 1:** Estimated annual type 2 diabetes (T2DM) incidence (per 1000 person-years) of adults aged 25–64 years in Samoa, by survey year

	1978	1991	1995	2002	2003	2010	2013
Men							
25–29	3.15	2.41	2.19	1.80	1.74	1.36	1.19
30–34	0.00^‡^	0.97	2.52	5.32	5.73	8.67	9.97
35–39	1.30	2.74	3.19	4.00	4.11	4.94	5.30
40–44	0.37	2.85	3.65	5.09	5.30	6.81	7.47
45–49	1.24	5.65	7.11	9.80	10.20	13.12	14.44
50–54	2.09	5.45	6.56	8.61	8.91	11.12	12.12
55–59	3.84	6.45	7.32	8.90	9.13	10.84	11.60
60–64	4.36	7.46	8.50	10.41	10.70	12.79	13.74
25–64^^^	1.12	3.61	4.43	5.92	6.14	7.73	8.44
95% CI (25-64)	0.76–1.48	3.04–4.18	3.73–5.13	5.39–6.45	5.24–7.03	7.00–8.45	7.73–9.15
Cumulative risk %^†^	1.24	3.34	4.02	5.25	5.43	6.73	7.30
95% CI (cumulative risk)	0.06–2.43	1.64–5.04	1.95–6.09	3.71–6.79	2.83–8.02	4.65–8.80	5.29–9.31
Women							
25–29	3.48	2.74	2.52	2.13	2.07	1.69	1.52
30–34	0.98	2.18	2.56	3.23	3.33	4.01	4.31
35–39	1.01	2.08	2.42	3.02	3.11	3.72	3.99
40–44	2.76	4.80	5.46	6.63	6.80	8.01	8.54
45–49	2.02	4.44	5.23	6.65	6.86	8.36	9.03
50–54	2.39	5.58	6.63	8.56	8.84	10.92	11.85
55–59	3.95	6.80	7.74	9.47	9.73	11.61	12.45
60–64	7.01	9.52	10.34	11.87	12.10	13.75	14.50
25–64^^^	2.55	4.08	4.58	5.48	5.61	6.57	7.00
95% CI (25-64)	2.05–3.04	3.52–4.64	3.90–5.26	5.00–5.97	4.83–6.40	6.04–7.11	6.48–7.53
Cumulative risk %^†^	2.33	3.74	4.20	5.03	5.15	6.02	6.41
95% CI (cumulative risk)	0.85–3.81	2.08–5.40	2.17–6.22	3.59–6.46	2.84–7.46	4.45–7.58	4.86–7.95
Samoa							
25–29	2.92	2.27	2.07	1.72	1.67	1.33	1.19
30–34	1.52	3.08	3.57	4.44	4.56	5.45	5.83
35–39	0.66	1.94	2.34	3.06	3.16	3.89	4.21
40–44	0.28	2.85	3.67	5.16	5.38	6.93	7.61
45–49	1.56	4.59	5.58	7.39	7.65	9.58	10.45
50–54	2.00	5.31	6.40	8.41	8.71	10.88	11.86
55–59	2.77	5.89	6.93	8.84	9.13	11.21	12.15
60–64	3.71	7.40	8.64	10.97	11.31	13.89	15.08
25–64^^^	1.74	3.62	4.24	5.36	5.52	6.72	7.25
95% CI (25–64)	1.44–2.04	3.24–4.00	3.76–4.71	5.01–5.70	4.95–6.10	6.45–6.99	6.84–7.67
Cumulative risk %^†^	1.53	3.28	3.84	4.88	5.03	6.12	6.61
95% CI (cumulative risk)	0.64–2.42	2.14–4.41	2.44–5.25	3.86–5.89	3.34–6.72	5.32–6.92	5.40–7.82

^^^Age-standardized to nearest previous censuses

^†^Cumulative risk over 25–64 years (Armitage et al. 2001)

^‡^Negative incidences were revised to zero. Incidence was calculated by the Stýblo birth cohort method (Morrell et al. 2016)

Projected national annual T2DM incidence in 2020 is estimated as 12.74 per 1000 person-years assuming that current period BMI increases (Table 2), equivalent to 957 new cases in 2020 based on the projected population of adults aged 25–64 years in 2020 of 75,045. This estimate is higher than the projected incidence based on period alone of 8.56 per 1000 person-years, equivalent to 643 new cases diagnosed in 2020.

**Table 2:** Projections of annual type 2 diabetes (T2DM) incidence (per 1000 person-years) of adults aged 25–64 years in Samoa in 2020 based on various body mass index (BMI) trend scenarios

	T2DM incidence and 95% CI in 2020		
	Men	Women	Samoa
2013 incidence^^^	8.44 (7.73–9.15)	7.00 (6.48–7.53)	7.25 (6.84–7.67)
Projected to 2020 (period trend)^^^	10.18 (9.40–10.95)	8.04 (7.48–8.60)	8.56 (8.11–9.01)
Projected to 2020^^†^ (including projected period BMI increases to 2020^‡^)	13.87 (12.96–14.77)	11.60 (10.77–12.42)	12.74 (11.87–13.60)
Projected to 2020: no BMI change 2013–2020 (baseline)^‡^	13.16 (12.28–14.04)	10.61 (9.82–11.41)	11.89 (11.06–12.73)
Projected from BMI change to 2020			
Weight loss 2013–2020^†^			
−1 kg	12.75 (11.88–13.61)	10.23 (9.45–11.01)	11.50 (10.67–12.32)
−2 kg	12.34 (11.49–13.20)	9.87 (9.10–10.63)	11.11 (10.30–11.92)
−3 kg	11.95 (11.11–12.79)	9.51 (8.76–10.26)	10.74 (9.94–11.54)
−4 kg	11.58 (10.75–12.40)	9.17 (8.43–9.91)	10.38 (9.60–11.16)
Weight gain 2013–2020^†^			
+1 kg	13.59 (12.69–14.48)	11.01 (10.20-11.81)	12.30 (11.45–13.16)
+2 kg	14.03 (13.12–14.94)	11.42 (10.60–12.24)	12.73 (11.86–13.60)
+3 kg	14.49 (13.56–15.41)	11.84 (11.01–12.68)	13.17 (12.29–14.05)
+4 kg	14.96 (14.02–15.90)	12.28 (11.43–13.13)	13.63 (12.73–14.52)

^^^Calculated from adjusted prevalences using birth cohorts from age-period (Lexis) matrix using the Stýblo method

^†^From Conway–Maxwell Poisson models using sex, age, and mean BMI (sex-specific), and age and mean BMI (national), including mean BMI as a dispersion regressor variable

^‡^Calculated using linear meta-regression based on eight surveys (Lin et al. 2016)

If population body weight in 2020 was maintained at 2013 levels, annual national T2DM incidence is projected to be 11.89 per 1000 person-years in 2020, equivalent to a reduction of 0.84 incident cases per 1000 per year compared to the number of cases based on the current period BMI trend, or 63 fewer new cases diagnosed in 2020. If population body weight could be reduced by 1 kg from 2013 levels, T2DM incidence in 2020 is estimated to be 11.50 per 1000 person-years, equivalent to preventing 1.24 new cases per 1000 persons annually (or 94 fewer new T2DM cases in 2020). A 4 kg reduction in mean weight results in an estimated T2DM incidence of 10.38 per 1000 person-years in 2020, a reduction of 2.36 new cases per 1000 annually (or 177 fewer new T2DM cases in 2020).

The deviances of the Poisson models without predictor variables (the null model) were 132.7 for men and 212.4 for women. With only age included, model deviance decreased to 33.9 for men and 24.7 for women. With addition of period, deviance further decreased to 12.4 for men and 9.4 for women. Alternatively, addition of birth cohort to the age-only model similarly reduced deviance to 10.5 for men and 8.1 for women, suggesting both period and cohort effects.

## Discussion

This study demonstrates the feasibility of deriving incidence trends from irregularly spaced sequential risk factor population prevalence surveys. It avoids the pitfalls of using other methods of calculating incidence, such as cohort studies, pharmaceutical registries, or compartment models, all of which have drawbacks from biases and generalisability, and may not be feasible in low resource countries.

Age explained much of the variation in deviance in regression models, and age-period and age-cohort analyses suggested similarity of period and birth cohort influences.

As a form of external validation, T2DM incidence calculated using the Stýblo method was compared to incidence derived from a previously conducted Samoan cohort study. Compared to incidence of T2DM found by McGarvey (2001) over 1991–1995 (5.3 per 1000 person-years in each sex), estimated national T2DM incidence rates in the present study for 1993 are similar for men (4.0 per 1000 person-years) and women (4.3 per 1000 person-years). The T2DM incidence estimates for Samoa are higher than the estimated Fiji Melanesian incidence rate of 2.3 per 1000 person-years (men) and 2.8 per 1000 person-years (women) in 1993 (Morrell et al. 2016), which is congruent with the higher T2DM prevalence in Samoa (2013: men 27.0%, women 22.6%) (Lin et al. 2016a) compared to Fiji Melanesians (2011: men 11.1%, women 13.6%) (Lin et al. 2016b). The present analysis has added to the previous incidence study in Fiji by provision of empirical cohort data in Samoa to enable external validation which also extends the method to a population with markedly higher levels of BMI and T2DM. Estimated incidence in 2013 in Samoa from the present study was 8.4 per 1000 person-years in men and 7.0 in women compared to Fiji Melanesians (for 2011) estimated to be approximately half that of Samoans at 4.1 per 1000 person-years in men and 4.7 in women (Morrell et al. 2016). The higher incidence found in Samoans may be due in part to the greater magnitude and longer duration of obesity in Samoans compared to Fiji Melanesians. Obesity (BMI ≥ 30 kg/m^2^) prevalence increased from 12.6 to 28.9% in Fiji Melanesian men and from 30.1 to 52.9% in women over 1980–2011 (Lin et al. 2016b), compared with increases from 23.5 to 53.1% in Samoan men and 43.7–73.4% in Samoan women over 1978–2013 (Lin et al. 2016a).

Our estimate of Samoan T2DM incidence for 1990–1994 and by McGarvey et al. (2001) for 1991–1995 was significantly lower than in neighbouring American Samoa, reported to be 28.7 per 1000 person-years in men and 21.7 in women for the same period, and derived also from a cohort study by McGarvey et al. (2001). The higher T2DM incidence in American Samoa likely relates, in part, to greater levels of obesity: the 2004 STEPS surveys in American Samoa found that 69% of men and 80% of women were obese (WHO 2007), compared to 53% (men) and 73% (women) in Samoa in 2013 (Lin et al. 2016a).

The recent Global Burden of Disease (GBD) 2015 study reported global diabetes incidence, estimated from statistical modeling, had increased by 30.6% between 2005 and 2015, but country-specific estimates were not provided (GBD 2015 Disease and Injury Incidence and Prevalence Collaborators 2016).

The higher incidence of T2DM in men found in the present investigation is congruent with findings from some other studies (Meisinger et al. 2002), but not all (Bonora et al. 2004). Cohort studies in Europe have found risk of T2DM incidence in men to be approximately 50% higher than in women (InterAct Consortium 2011). At a given BMI, men tend to have higher levels of visceral and hepatic adipose tissue deposits compared to women (Geer and Shen 2009), which is associated with increased insulin resistance and T2DM (Carey et al. 1997). It is also possible that oestrogen has a protective effect in younger women by increasing insulin sensitivity and reducing insulin resistance, although insulin sensitivity declines after menopause (Geer and Shen 2009).

In some countries such as the US (from surveys using self-report) (Centers for Disease Control 2015), incidence of T2DM has fallen in recent years, indicating either that the pool of genetic susceptibles has been exhausted and population threshold has been reached, or cumulative population health messages have begun to take effect, since adult obesity (BMI ≥ 30 kg/m^2^) in the US has plateaued in men at 35%, but not women, since 2005 (Flegal et al. 2016). In Samoa, our modeling of T2DM incidence suggests that a plateau will not be reached in the short term.

Increases in premature adult mortality from non-communicable diseases (NCD) are slowing or halting life expectancy increases in Fiji (Taylor et al. 2013), probably in Tonga (Hufanga et al. 2012), and possibly in Samoa. Estimates from the 2015 GBD study suggest that life expectancy in Samoan women has made only small gains: from 73 to 74 years between 1990 and 2015; whereas estimates for Samoan men have increased from 66 to 72 years over the same period (Institute for Health Metrics and Evaluation 2016), although incomplete mortality reporting makes all estimates fraught. As demonstrated by this study, small changes in mean population weight may have modest effects in reducing the number of new T2DM cases diagnosed in future.

However, the design and content of population weight loss interventions for Samoa are not clear. Initiatives that have successfully demonstrated weight loss and consequent reductions in T2DM incidence are limited to trials involving volunteers with pre-diabetes (Diabetes Prevention Program Research Group 2002). These studies have involved intensive resource inputs and it is yet to be seen if less intensive population-based interventions can lower weight and reduce T2DM incidence (American Diabetes Association 2004). Recently, some governments have introduced taxes on unhealthy food and drink to reduce population weight (Batis et al. 2016). While the early results indicate reductions in consumption, this has yet to be translated into population weight loss (Batis et al. 2016). Effective interventions to lower weight and consequent T2DM morbidity and mortality are required without delay in Samoa and other low resource countries where T2DM incidence is likely increasing. To assess effectiveness of such interventions requires incidence data which are not readily available.

### Conclusions

The methods used in this study demonstrate the feasibility of calculating T2DM incidence from irregularly conducted population risk factor prevalence surveys, which produce estimates of incidence similar to estimates calculated from cohort studies. The technique outlined may be applied to other low resource settings especially where other methods of calculating incidence are not feasible, and in high income countries to complement other methods of monitoring T2DM population incidence.

## Electronic supplementary material

Below is the link to the electronic supplementary material.

Supplementary material 1 (DOCX 16 KB)
