# Supplementary material for: Diabetes incidence and projections from prevalence surveys in Samoa over 1978–2013
Source: Int J Public Health. 2017 Mar 9;62(6):687–94. doi: 10.1007/s00038-017-0961-x (PMC5487887; doi:10.1007/s00038-017-0961-x)
Supplement: Supplementary file 1 — Supplementary material 1 (DOCX 16 KB) [file 38_2017_961_MOESM1_ESM.docx]

**Appendix**

Diabetes incidence and projections from prevalence surveys in Samoa over 1978-2013

S. Lin, T. Naseri, C. Linhart, S. Morrell, R. Taylor, S.T. McGarvey, D.J. Magliano, P. Zimmet

Table 1. Designation of type 2 diabetes in included Samoan surveys.

| Survey | Input blood sample | Definition | Notes |
| --- | --- | --- | --- |
| 1978 NCDRF | Venous plasma | FPG ≥7.0mmol/L and/or on T2DM medication |  |
| 1991 NCDRF | Venous plasma | FPG ≥7.0mmol/L and/or on T2DM medication |  |
| 1991 SACRF | Venous serum | FPG ≥7.0mmol/L and/or on T2DM medication | Surveys adjusted to include estimates of known T2DM cases as these participants were excluded from original study. |
| 1995 SACRF | Venous serum | FPG ≥7.0mmol/L and/or on T2DM medication |  |
| 2002 STEPS | Capillary whole blood | FBG ≥6.1mmol/L and/or on T2DM medication | Glucose was measured using a glucose meter that produced measurements in whole blood concentrations |
| 2003 SFSOD | Venous serum | FPG ≥7.0mmol/L and/or on T2DM medication | Serum glucose measurements were converted to plasma equivalent using the following formula: *-0.137+1.047x*, where x is serum glucose (mmol/L) (Rydén et al. 2007) |
| 2010 GWAS | Venous serum | FPG ≥7.0mmol/L and/or on T2DM medication |  |
| 2013 STEPS | Capillary whole blood | FPG ≥7.0mmol/L and/or on T2DM medication | Glucose was measured using a plasma calibrated glucose meter. |

Included surveys: 1978 and 1991 Non-communicable disease risk factor (NCDRF) (Zimmet et al. 1981; Collins et al. 1994); 1991-95 Samoan Adiposity and Cardiovascular Risk Factor (SACRF) (McGarvey 2001; Ezeamama et al 2006); 2002 and 2013 STEPS (WHO 2008, 2014); 2003 Samoa Family Study of Overweight and Diabetes (SFSOD) (DiBello et al. 2009); 2010 Genome-Wide Association Study (GWAS) (Hawley et al. 2014).

FBG = fasting whole blood glucose; FPG = fasting plasma glucose; T2DM = type 2 diabetes.

Full details of T2DM designation have been previously published in Lin et al. 2016.

REFERENCES

Collins VR, Dowse GK, Toelupe PM, et al (1994) Increasing prevalence of NIDDM in the Pacific island population of Western Samoa over a 13-year period. Diabetes Care 17:288-96

DiBello JR, Baylin A, Viali S, Tuitele J, Bausserman L, McGarvey ST (2009) Adiponectin and T2DM in Samoan adults. Am J Hum Biol 21:389-391

Ezeamama AE, Viali S, Tuitele J, McGarvey ST (2006) The influence of socioeconomic factors on cardiovascular disease risk factors in the context of economic development in the Samoan archipelago. Soc Sci Med 63:2533-2545

Hawley NL, Minster RL, Weeks DE, et al (2014) Prevalence of adiposity and associated cardiometabolic risk factors in the Samoan Genome-Wide Association Study. Am J Hum Biol 26:491-501

Lin S, Naseri T, Linhart C, et al (2016a) Trends in diabetes and obesity in Samoa over 35 years, 1978-2013. Diabet Med doi: 10.1111/dme.13197. [Epub ahead of print]

McGarvey ST (2001) Cardiovascular disease (CVD) risk factors in Samoa and American Samoa, 1990-1995. Pac Health Dialog 8:157-162

Rydén L, Standl E, Bartnik M, et al (2007) Guidelines on diabetes, pre-diabetes, and cardiovascular diseases: executive summary. The Task Force on Diabetes and Cardiovascular Diseases of the European Society of Cardiology (ESC) and of the European Association for the Study of Diabetes (EASD). Eur Heart J 28:88-136

Zimmet P, Faaiuso S, Ainuu J, Whitehouse S, Milne B, DeBoer W (1981) The prevalence of diabetes in the rural and urban Polynesian population of Western Samoa. Diabetes 30:45-51

WHO (2008) Samoa NCD Risk Factors STEPS report 2002. World Health Organization, Apia

WHO (2014) Samoa NCD Risk Factors STEPS report 2013. World Health Organization, Apia
